# Editorial: Targeting adipose tissue for the treatment of metabolic alterations

**DOI:** 10.3389/fphar.2026.1797423

**Published:** 2026-02-11

**Authors:** Lorenzo Flori, Bruno Ramos-Molina, Vincenzo Calderone, Alma Martelli

**Affiliations:** 1 Department of Pharmacy, University of Pisa, Pisa, Italy; 2 Obesity, Diabetes, and Metabolism Laboratory, Biomedical Research Institute of Murcia (IMIB), Murcia, Spain; 3 Interdepartmental Research Centre of Ageing, Biology and Pathology, University of Pisa, Pisa, Italy; 4 Center for Instrument Sharing of the University of Pisa (CISUP), University of Pisa, Pisa, Italy

**Keywords:** adipogenesis, adipose tissue, AMPK, browning, crosstalk, energy expenditure, metabolic disorders, thermogenesis

The global increase in obesity and related metabolic disorders is one of the most significant biomedical challenges of the twenty-first century (Rosen and Spiegelman, 2014). Adiposopathy, described both as a quantitative increase in the deposition of white adipose tissue (WAT) and a qualitative phenotypic alteration, is now recognized as a driver of systemic metabolic dysregulation, including insulin resistance, type 2 diabetes, metabolic dysfunction-associated steatotic liver disease (MASLD), and cardiovascular disease (Longo et al., 2019) (Li et al.). In this context, adipose tissue has emerged as a key regulatory organ whose remarkable plasticity provides valuable therapeutic potential (Castellano-Castillo et al., 2020). The Research Topic “*Targeting Adipose Tissue for the Treatment of Metabolic Alteration*” brings together experimental and translational studies that collectively demonstrate how modulating adipose tissue biology, particularly regarding adipogenesis, browning and thermogenesis, can be utilized to restore metabolic homeostasis.

Adipose tissue exists along a functional continuum, ranging from energy storage of the WAT to thermogenic induction on brown adipose tissue (BAT), with beige adipocytes representing an inducible intermediate phenotype. The ability of WAT or preadipocytes precursors to undergo browning and acquire brown-like features has attracted considerable attention as a strategy to increase energy expenditure and improve metabolic health (Ghesmati et al., 2024; Flori et al., 2023a). In parallel, growing evidence underscores the importance of adipose tissue crosstalk with the nervous system, endocrine organs, muscle, liver, and immune system in shaping systemic metabolic outcomes (Cheong and Xu, 2021; Jia et al., 2024).

Two opinion articles provide the framework for this Research Topic by challenging conventional views on obesity treatment and highlighting emerging paradigms on adipose tissue research. The first introduces the concept of physiological resilience in brown-white adipose tissue dynamics, emphasizing that the therapeutic potential of adipose tissue lies not only in its mass or activity, but in its intrinsic ability to adapt to environmental, metabolic, and energetic issues (Zayachkivska et al.). This perspective reframes obesity management as a problem of impaired plasticity rather than simple energy imbalance. The second opinion advances a translational perspective that bridges basic discovery and clinical application of how strategies targeting adipose tissue may modulate metabolic and immune pathways simultaneously (Chávez-Jiménez et al.). By proposing dual-use agents with both anti-obesity and antiviral activity, this contribution underscores the convergence of metabolic regulation and host defense mechanisms within adipose tissue biology.

Several original research articles directly address the molecular and cellular mechanisms driving adipose tissue browning and thermogenesis. In a high-fat diet-induced obesity model, transcriptomic and ultrastructural analyses demonstrate that pharmacological intervention with the traditional Chinese medicine formulation “Dahuang-Huanglian” induces metabolic improvements, including reduced adiposity, enhanced insulin sensitivity, and increased number of mitochondria in adipocytes. These effects are mechanistically linked to the upregulation of thermogenesis-related genes and activation of the AMPK/SIRT1/PGC-1α axis, together with increased expression of canonical browning markers such as UCP1, PRDM16, and PPARγ (Zhang et al.). This work provides compelling evidence that targeting intracellular energy related adipocytes pathways can promote WAT browning and counteract diet-induced metabolic dysfunction.

Complementary findings emerge from studies focusing on BAT activation as a therapeutic strategy for the management of metabolic syndrome. Administration of “Xiasangju”, Chinese medicine formulation composed of *Prunellae Spica* (*Prunella vulgaris* L.), mulberry (*Morus alba* L.) leaf and *Flos Chrysanthemi Indici* (*Chrysanthemum indicum* L.), was shown to enhance noradrenaline biosynthesis, activate BAT thermogenesis, and improve glucose and lipid metabolism in a model of metabolic syndrome. Notably, this intervention also modulated gut microbiota composition and strengthened intestinal barrier integrity, leading to reduced systemic inflammation and preserved catecholaminergic signaling to BAT (He et al.). These results underscore the integrative nature of adipose tissue regulation, linking thermogenesis to neuroimmune and gut-adipose axes.

The transcriptional control of adipocyte phenotype is further explored in both *in vitro* and *in vivo* models. A detailed and reproducible differentiation protocol using 3T3-L1 cells defines the temporal acquisition of beige adipocyte features and identifies rosiglitazone, a PPARγ agonist, as a robust reference compound for inducing “*de novo*” browning. This study provides an essential methodological framework for this field and reinforces the central role of PPARγ signaling in adipocyte plasticity (Flori et al.). In parallel, mechanistic work using the traditional Chinese medicine formulation “Ling-gui-zhu-gan” demonstrates that adipocyte browning can be regulated at the post-transcriptional level through suppression of miR-27b and subsequent activation of the PRDM16-dependent thermogenic Pathway (Ye et al.). Together, these studies highlight both transcriptional and microRNA-mediated mechanisms as viable targets for modulating adipose tissue phenotype.

Beyond adipocyte mechanisms, several contributions emphasize the role of neural and endocrine regulation in shaping adipose tissue function. Genetic inactivation of the imprinted gene Mest in female mice resulted in protection against diet-induced obesity, driven by increased spontaneous physical activity and enhanced non-exercise activity thermogenesis. Mechanistically, these effects were associated with altered hypothalamic thyroid hormone signaling, illustrating how central regulation of behavior and energy expenditure feeds back onto adipose tissue remodeling (Anunciado-Koza et al.). Similarly, modulation of Gi/o-coupled receptor signaling in SNAP25Δ3 mice revealed pronounced sex-specific differences in metabolic protection, adipose tissue browning, and insulin sensitivity. Strikingly, female mice maintained metabolic protection even under thermoneutral conditions and independently of ovarian hormones, suggesting the existence of sex-specific, hormone-independent mechanisms controlling adipose tissue thermogenesis (Young et al.).

The translational relevance of adipose tissue targeting is reinforced by integrative omics approaches applied to human samples. Proteomic and metabolomic profiling of visceral adipose tissue from patients with obesity undergoing bariatric surgery identified distinct molecular signatures associated with lipid droplet biology, AMPK signaling, cortisol biosynthesis, and insulin resistance. The integration of protein-metabolite networks revealed candidate regulators of postoperative metabolic improvement, emphasizing the value of multi-omics strategies for uncovering novel therapeutic targets within adipose tissue (Li et al.).

Importantly, the Research Topic also addresses metabolic disease heterogeneity and points out adipose tissue as a mediator across diverse clinical conditions. A comprehensive meta-analysis comparing lean and women with obesity and polycystic ovary syndrome (PCOS) revealed substantial differences in lipid profiles, insulin resistance, and blood pressure, underscoring the contribution of adiposity to the metabolic burden of PCOS and the need for tailored therapeutic approaches (Zheng et al.). Population-based data further demonstrate that adipose tissue quantity and distribution regulate the inverse relationship between muscle quality and hyperuricemia, highlighting adipose tissue as a key connection point between musculoskeletal health and metabolic risk (Shao et al.).

Rare disorders characterized by adipose tissue dysfunction (Flori et al., 2023b) further expand the therapeutic scope of this Research Topic. In congenital generalized lipodystrophy, bioinformatic analyses identified immune-related chemokine receptors as diagnostic biomarkers and therapeutic targets. Experimental validation showed that ibuprofen-mediated targeting of CXCR family members improved glucose tolerance, lipid metabolism, and hepatic steatosis, highlighting immune-adipose interactions as viable pathways in metabolic disease even in the context of adipose tissue deficiency (Cao et al.).

Taken together, the contributions to this Research Topic converge on a unifying conclusion: adipose tissue plasticity is a central aspect of metabolic health. The studies presented that metabolic outcomes can be favorably influenced by acting on adipose tissue at multiple levels, by promoting browning and thermogenic activation, by modulating the neuroendocrine circuits that govern energy expenditure and adipose tissue remodeling, and by redefining adipose tissue as an active mediator of systemic metabolic risk across diverse clinical contexts. Rather than representing isolated strategies, these approaches are complementary facets of a broader therapeutic paradigm in which adipose tissue is a dynamic and integrative target for the prevention and treatment of metabolic disorders.

In conclusion, this Research Topic position establishes adipose tissue not merely as a passive target but as an active regulator of metabolic resilience. By integrating insights from molecular biology, physiology, and clinical research, the studies presented here advance the understanding of how adipogenesis, browning, and thermogenesis can be strategically used to counteract metabolic alterations. Together, they provide a strong rationale to endure in investigation of adipose tissue, core of interventions in the prevention and treatment of metabolic disease.
